# Knowledge of prostate cancer among males attending a urology clinic, a South African study

**DOI:** 10.1186/s40064-015-0824-y

**Published:** 2015-02-10

**Authors:** Nathaniel Mofolo, Olwethu Betshu, Ogomoditse Kenna, Sarah Koroma, Tlalane Lebeko, Frederik M Claassen, Gina Joubert

**Affiliations:** Department of Family Medicine, Faculty of Health Sciences, University of the Free State, 205 Nelson Mandela Drive, Bloemfontein, South Africa; Medical students, Faculty of Health Sciences, University of the Free State, Bloemfontein, South Africa; Department of Urology, Faculty of Health Sciences, University of the Free State, Bloemfontein, South Africa; Department of Biostatistics, Faculty of Health Sciences, University of the Free State, Bloemfontein, South Africa

**Keywords:** Prostate, Cancer, Knowledge, South Africa, Outpatient

## Abstract

**Background:**

In South Africa, the rate of histologically diagnosed prostate cancer is 40.1 per 100 000 in whites and 14 per 100 000 in blacks. However, blacks have limited access to diagnostic facilities and present late with an advanced disease. Knowledge about prostate cancer in the South African male population is necessary in order to increase the acceptance of early prostate cancer screening.

**Objective:**

This study assessed the knowledge of prostate cancer among men attending the urology outpatient clinic at a tertiary hospital in South Africa.

**Methods:**

A cross-sectional study was conducted from February to March 2010. A structured questionnaire was administered to participants using consecutive sampling of eligible patients and consisted of sections on sociodemographic details and knowledge about prostate cancer. A total of 346 males, 35 years of age and older, participated in the study.

**Results:**

The majority of the respondents (n = 258; 75.0%) were black, married (n = 220; 64.0%), from the Free State Province (n = 320; 92.8%), and had access to television (n = 248; 71.7%). Only 38 (11.0%) knew the three main symptoms and signs associated with prostate cancer. Level of school education, race and language were statistically significantly associated with level of knowledge whereas age and marital status were not.

**Conclusion:**

More than half (54.4%) of the respondents had not heard of prostate cancer. The majority of men who had heard of prostate cancer had a moderate level of knowledge. The factors significantly associated with level of knowledge need to be considered in educational campaigns, prostate cancer screening and treatment.

## Background

Prostate cancer is one of the most common cancers affecting the male population globally. It is rated the second leading cause of cancer death among males in the USA, and the most commonly diagnosed non-cutaneous malignancy (Ali and Ali 2008; Pruthi et al. 2006). In Southern African men, prostate cancer has an incidence of 40.5 per 100 000 of the population per year, and a mortality rate of 22.5 per 100 000 per year (Parkin et al. 2005). In South Africa, prostate screening is recommended across all men from age of 45 years onwards in the absence of identifiable risk factors (South African Prostate Cancer Foundation, 2013 Guidelines). The risk of getting prostate cancer increases after 50 years of age, with the annual incidence being the highest in men older than 70 years of age (Goddard et al. 2006). Men with a family history of prostate cancer have an increased risk to develop the disease (Pruthi et al. 2006).

It is not clear whether an increase in the utilisation of prostate cancer screening services might be associated with actual or perceived knowledge of prostate cancer (Agho and Lewis 2001). An American study found a positive relationship between low-level income and low levels of knowledge about prostate cancer, which was attributed to poor access to information sources (Deibert et al. 2007). It was further found that low levels of education, older age and speaking a non-English language were directly related to poor knowledge about the disease. The same study reported that men involved in programmes and seminars on prostate cancer and treatment did not have more knowledge about prostate cancer compared to those who were not participating in such programmes. Another study also concluded that physicians contribute towards the level of patients’ knowledge about prostate cancer, but a greater number of participants had never discussed anything related to prostate cancer and screening tests with their physicians (Smith et al. 1997).

In contrast, an African American study found that the higher the level of school education and income, the lesser the knowledge of prostate cancer (Agho and Lewis 2001).

South African studies focused more on the prevalence of the disease, knowledge about the screening methods or participation in screening surveys (Heyns et al. 2003; Walker and Halse 1999; Walker 1986). Not many studies have been done in Africa in assessing knowledge of prostate cancer, and the study that has been done also had the same focus as South African studies (Ajape et al. 2010). In South Africa, little is known about the level of knowledge about prostate cancer in the male population. We report on a survey done on men attending an outpatient urology clinic at the Bloemfontein Academic Hospital complex. The study also investigated the association of factors such as age, marital status, race, education and language, with the level of knowledge about prostate cancer.

## Methods

This cross-sectional study was conducted among men 35 years of age and older attending the outpatient urology clinic at a tertiary Academic Hospital in South Africa, Free State Province, Bloemfontein Academic Hospital Complex. The Bloemfontein Academic Hospital Complex offers healthcare services largely to the Free State community, with some drainage areas which mainly include the Northern and Eastern Cape provinces, and Lesotho.

A target population of 400 men was set, so as to enable subgroup comparisons, and 346 men were enrolled in the survey. The survey was conducted from February to March 2010. Consecutive male patients attending the clinic during weekdays were included in the study. Critically ill patients and those who could not understand English, Afrikaans or Sesotho were excluded. A questionnaire was compiled in the three languages most commonly spoken in the region, namely English, Afrikaans and Sesotho. It should be mentioned that there is no word for ‘prostate’ in Sesotho, consequently more extensive explanation had to be given to Sesotho-speaking participants.

Participation in the study was voluntary. Questionnaires were self-completed anonymously by participants. In cases where the researchers had to assist with clarification of questions or filling in of questionnaire, written informed consent was obtained as required by the Ethics Committee of the Faculty of Health Sciences. The questionnaire consisted of sections to acquire demographic details and specific questions related to participants’ knowledge of prostate cancer. The race of a respondent was based on the patient’s own feedback. When a respondent indicated that he had never heard of prostate cancer, the interview was terminated and only the demographic information of these individuals was used. The questionnaire also enquired with regard to the medium of acquisition of knowledge on prostate cancer.

The questionnaire had 14 items related to knowledge of prostate cancer. The level of knowledge was categorised as low (0–4), moderate (5–9) or high (10+), based on the total number of correct answers. A score of zero to four correct answers was regarded as a low level of knowledge, five to nine correct answers as a moderate level of knowledge, and ten or more correct answers as a high level of knowledge.

The Ethics Committee of the Faculty of Health Sciences, University of the Free State (UFS), and the chief executive officer of the Universitas Academic Complex approved the study. The findings of a pilot study involving 25 patients were included in the final results after the data was validated and upon advice from the biostatistician.

All results were summarised categorically by frequencies and percentages. The univariate associations between factors were investigated using chi-squared or Fisher’s exact tests, in the case of sparse cells. Multivariate analysis was conducted on factors found to associate significantly with level of knowledge on univariate analysis.

## Results

The characteristics of the participants in the study are summarised in Table 1. The level of knowledge among participants is presented in Table 2. The number of men that could correctly answer questions related to knowledge of prostate cancer is presented in Table 3.

**Table 1:** **Characteristics of participants**

**Variable**	**Frequency**	**Percentage**
Race (n = 344)*		
Black	258	75.0
Caucasian	55	16.0
Coloured	26	7.6
Asian	5	1.4
***Age group (n = 344)***		
35–44 years	112	32.4
45–54 years	95	27.5
55–64 years	86	24.9
65–74 years	39	11.3
≥75 years	12	4.0
***Language*** ^***#***^ ***(n = 346)***		
Afrikaans	243	70.2
English	199	57.5
Sesotho	254	73.4
isiZulu	65	18.8
***Marital status (n = 344)***		
Married	220	64.0
Unmarried	89	25.9
Divorced	35	10.2
***Area of residence (n = 345)***		
Free State	320	92.8
Northern Cape	13	3.8
Eastern Cape	5	1.4
Lesotho	5	1.4
North West	1	0.3
Other	1	0.3
***Level of school education (n = 346)***		
None	34	9.8
Primary	128	37.0
Secondary	148	42.8
Tertiary	36	10.4
***Employment (n = 344)***		
Unemployed	103	29.9
Employed	86	25.0
Self-employed	35	10.2
Pensioner	118	34.3
Student	2	0.6
***Type of media exposure (n = 346)***		
Television	248	71.7
Radio	231	66.8
Newspapers	116	33.5
Posters	54	15.6
Magazines	41	11.8
Internet	23	6.6
None	7	2.0

(n = 346).

*Number of participants who provided information on each variable.

^#^More than half of the participants were multilingual.

**Table 2:** **Level of knowledge of prostate cancer of participants who had heard of prostate cancer according to demographic variables**

**Variable**	**Level of knowledge (number of correct answers)**						**p-value**
	**Low (0–4)**		**Moderate (5–9)**		**High (10–14)**		
	**n**	**%**	**N**	**%**	**n**	**%**	
***Race***							
Black (n = 102)	26	25.5	73	71.6	3	2.9	0.0596
Caucasian (n = 39)	4	10.3	30	76.9	5	12.8	
Coloured (n = 12)	2	16.7	9	75.0	1	8.3	
***Age group***							
35–44 years (n = 44)	11	25.0	31	70.5	2	4.5	0.1736
45–54 years (n = 44)	13	29.6	29	65.9	2	4.5	
55–64 years (n = 41)	8	19.5	31	76.6	2	4.9	
65–74 years (n = 25)	1	4.0	21	84.0	3	12.0	
≥75 years (n = 4)	0	0	4	100	0	0	
***Language***							
Afrikaans (n = 120)	23	19.2	88	73.3	9	7.5	0.4509
English (n = 106)	19	17.9	81	76.4	6	5.7	
Sesotho (n = 101)	24	23.7	74	73.3	3	3.0	
isiZulu (n = 35)	9	25.7	22	62.9	4	11.4	
***Marital status***							
Married (n = 111)	7	24.1	21	72.4	1	3.5	0.8854
Unmarried (n = 29)	23	20.7	80	72.1	8	7.2	
Divorced (n = 17)	3	17.7	14	82.3	0	0	
***Level of school education***							
None (n = 5)	0	0	5	100	0	0	0.0335
Primary (n = 45)	14	31.1	31	68.9	0	0	
Secondary (n = 80)	17	21.2	56	70.0	7	8.8	
Tertiary (n = 28)	2	7.1	24	85.7	2	7.1	
***Employment***							
Unemployed (n = 41)	14	34.2	27	65.8	0	0	0.0290
Employed (n = 40)	7	17.5	30	75.0	3	7.5	
Self-employed (n = 19)	6	31.6	11	57.9	2	10.5	
Pensioner (n = 55)	6	10.9	45	81.8	4	7.3	
***Screening test done***							
Yes (n = 43)	4	9.3	36	83.7	3	7.0	0.1109
No (n = 109)	26	23.9	77	70.6	6	5.5	

(n = 158).

**Table 3:** **Individual questions on prostate cancer answered correctly by participants who had heard of prostate cancer**

**Questions**	**Correct answers provided**	
	**n**	**%**
Where is the prostate located?	140	88.6
What causes prostate cancer?		
*Family inheritance*	51	32.3
*Diet*	22	13.9
*Both correct and no incorrect options chosen*	10	6.3
What are the risk factors?	91	57.6
How can prostate cancer be prevented?		
*Exposure to sunlight*	11	7.0
*Certain vitamins*	47	29.8
*Both correct and no incorrect options chosen*	1	0.6
How can prostate cancer spread?	116	73.4
What are the signs and symptoms of prostate cancer		
*Blood in urine*	46	29.1
*Difficulty passing urine*	85	53.8
*Weight loss*	33	20.9
*All three correct and no incorrect options chosen*	18	11.4
Can prostate cancer be cured?	123	77.9
Does it affect both men and women?	92	58.2
At what age should screening for prostate cancer commence?	39	24.7
Where can screening services be provided?	129	81.7

(n = 158).

Overall, less than half (n = 158; 45.7%) of the respondents indicated that they had heard of prostate cancer. Analysed by race group, 70.9% of white, 46.2% of coloured and 39.5% of black respondents had heard of prostate cancer (p < 0.0001). Of those who had heard of prostate cancer, 20.9% had a low level of knowledge, 73.4% a moderate level and 5.7% a high level of knowledge (Table 2). The media to which respondents were most commonly exposed were television (71.7%) and radio (66.8%) (Table 1).

Level of knowledge was not statistically significantly associated with age, language, marital status or whether the patient had previously undergone prostate cancer screening (Table 2). Most men had a moderate level of knowledge, which was found to increase with level of school education. Black respondents had the highest percentage of low level knowledge, but this difference was not statistically significant (p = 0.06) (Table 2). Pensioners showed the highest level of moderate knowledge (81.8%), while the highest level of low knowledge was found among the unemployed (34.2%) (Table 2). There was a statically significant association between race and level of school education (p < 0. 0001) and age and level of school education (p < 0.01) (Table 2). In a multivariate analysis considering race, age group and education level as possible factors associated with level of knowledge only education level was statistically significant (p = 0.03) (Table 2).

School education was not associated with previous prostate cancer screening (p = 0.46), whereas the association between age and the previous use of prostate cancer screening was statistically significant (p < 0.0001) with only 8.7% of the age group 35–44 years having had previous prostate cancer screening at any health facility compared to 67% of those 65 years and older (Table 2).

## Discussion

The majority of participants (59.9%) were below the age of 55 years, and only 15.3% were 65 years or older. There is thus an over representation in the study of men at the lowest risk of prostate cancer.

It is disturbing that only 46% of the men aged 35 and above had heard of prostate cancer. From the results it is clear that, in general, most men (73.4%) who had heard of prostate cancer had a moderate level of knowledge of the disease. When interpreting our findings it must be kept in mind that the high knowledge category (10/14 to14/14 questions answered correctly) cover the range from 71–100%, which may be considered by other researchers as an over estimation of knowledge. On univariate analysis the level of knowledge about prostate cancer showed a statistically significant association with the level of school education and employment, and close to significant with race, but not with age, language, marital status and previous prostate cancer screening. Only 18 men (11.4%) knew the three main symptoms and signs of the disease and only 39 (24.7%) knew the age at which prostate screening becomes important (Table 3). In South Africa, prostate screening involves both Digital Rectal Examination (DRE) and Prostate Specific Antigen (PSA), and is recommended in males from 40 years of age in black South African if there is family history of prostate cancer and or breast cancer in the first degree relative, and generally from 45 years onwards from all other males (The Prostate Cancer Foundation of South Africa; 2013).

The possible reason for participants’ good knowledge regarding the location of the prostate is that the Sesotho translation used in the questionnaire to refer to prostate and prostate cancer could have indicated to participants that the prostate is located in the pelvic area.

This study identified a considerable level of poor knowledge of prostate cancer among black African men, which leads to similar findings and conclusion with other African studies done in Uganda and Burkina Faso (Nakandi et al. 2013 and Kabore et al. 2014). The level of education was also found to have strong correlation with high level of knowledge of prostate cancer (Kabore et al. 2014). A systematic review of the literature on perceptions of prostate cancer among black African and black Caribbean men also found poor knowledge of prostate cancer across all groups (Pedersen et al. 2012). In South Africa, prostate cancer is equally common across all race groups (South African Prostate Cancer Foundation, 2013). This supports our findings that indicate that prostate cancer awareness needs to be increased in all men regardless of race.

The challenges regarding appropriate terminology in different languages must be taken into account to optimise education and awareness campaigns. In this study, television and radio were shown to be the most available media. Health promotion, education and prevention using these media, therefore become key strategies to raise awareness, especially from men of 40 years onwards and those who have family history of prostate cancer. It will also be important to target men from rural areas, poor socio-economic background and with low level of education, as it was observed from Universitas Academic Hospital that these men present late with an advanced disease. More follow up scientific studies are needed in South Africa to determine the accessibility of health services including prostate screening, reasons for late referral especially among black Africans and knowledge of risk factors. Education and screening can be done at primary health care level to increase the level of awareness in the community. It is also important to include both patients and physicians in these awareness campaigns, in order to create culturally relevant prostate cancer information and testing services.

### Limitations

The patients’ diagnoses at the time of the study were not taken into consideration so it was therefore not known whether any of them had been previously diagnosed with prostate disease. The number of times the patient attended the clinic was also not taken into consideration even though duplication of participants was avoided. The study was limited only to men attending outpatient urology clinics, and therefore evaluation of the knowledge among hospitalised and critically ill patients and the general population was not included in the survey. The study did not include men who have health insurance. The lack of an African word for ‘prostate’ might have given away the answer about the location of prostate in the questionnaire to those participants that needed more explanation.

A further limitation is that certain aspects of knowledge were not assessed, for example, that prostate cancer can present without symptoms.

## Conclusion

More than half of the respondents indicated that they had not heard of prostate cancer. Of those who have heard of the condition, the majority had moderate knowledge. In multivariate analysis level of school education was significantly associated with knowledge. Factors significantly associated with level of knowledge need to be considered in educational campaigns, prostate cancer screening and treatment.
